# Versatile microscale screening platform for improving recombinant protein productivity in Chinese hamster ovary cells

**DOI:** 10.1038/srep18016

**Published:** 2015-12-11

**Authors:** Henning Gram Hansen, Claes Nymand Nilsson, Anne Mathilde Lund, Stefan Kol, Lise Marie Grav, Magnus Lundqvist, Johan Rockberg, Gyun Min Lee, Mikael Rørdam Andersen, Helene Faustrup Kildegaard

**Affiliations:** 1 The Novo Nordisk Foundation Center for Biosustainability, Technical University of Denmark (DTU), Hørsholm, Denmark; 2 Department of Systems Biology, Technical University of Denmark (DTU), Lyngby, Denmark; 3 Department of Proteomics and Nanobiotechnology, Royal Institute of Technology (KTH), Stockholm, Sweden; 4 Department of Biological Sciences, KAIST, Daejeon, Republic of Korea

## Abstract

Chinese hamster ovary (CHO) cells are widely used as cell factories for the production of biopharmaceuticals. In contrast to the highly optimized production processes for monoclonal antibody (mAb)-based biopharmaceuticals, improving productivity of non-mAb therapeutic glycoproteins is more likely to reduce production costs significantly. The aim of this study was to establish a versatile target gene screening platform for improving productivity for primarily non-mAb glycoproteins with complete interchangeability of model proteins and target genes using transient expression. The platform consists of four techniques compatible with 96-well microplates: lipid-based transient transfection, cell cultivation in microplates, cell counting and antibody-independent product titer determination based on split-GFP complementation. We were able to demonstrate growth profiles and volumetric productivity of CHO cells in 96-half-deepwell microplates comparable with those obtained in shake flasks. In addition, we demonstrate that split-GFP complementation can be used to accurately measure relative titers of therapeutic glycoproteins. Using this platform, we were able to detect target gene-specific increase in titer and specific productivity of two non-mAb glycoproteins. In conclusion, the platform provides a novel miniaturized and parallelisable solution for screening target genes and holds the potential to unravel genes that can enhance the secretory capacity of CHO cells.

The production of recombinant therapeutic proteins is a rapidly growing industry with a global sales value of approximately $140 billion in 2013^1^. The main production host of biopharmaceuticals is Chinese hamster ovary (CHO) cells^2^, the productivity of which has improved substantially since the mid-1980s with focus on media composition and bioprocess design^3^,^4^. Production of therapeutic monoclonal antibodies (mAbs) in CHO cells in optimized bioprocesses typically reaches 5 g/L titers^5^ and specific productivity (*q*_p_) of 50–90 pg per cell per day (pcd)^3^ resulting in production costs accounting for only 1 to 5% of the sales price^5^. However, significantly lower titers and *q*_p_ are more common for glycosylated non-mAb biopharmaceuticals^5^,^6^. Thus, increasing titer and *q*_p_ for non-mAb glycoproteins holds the potential to reduce production costs significantly.

Genetic engineering of CHO host cells is a well-established method for improving productivity by enhancing cell survival and *q*_p_ or increasing cell growth and *q*_p_^7^. Several studies have demonstrated positive effects of overexpressing genes on productivity of recombinant glycoproteins in CHO cells (for reviews see^7^,^8^). However, contradictory effects on *q*_p_ have been reported for some genes^8^,^9^. These discrepancies are likely a combined effect of multiple factors. For example, when working with stable clones, observed effects may be a consequence of clonal variation^6^,^10^. Moreover, the effects of ectopic expression of recombinant genes likely depend on physicochemical characteristics of the therapeutic glycoprotein^9^. A transient transfection-based overexpression screening platform has the potential to address these issues, because it enables complete interchangeability of which genes (hereafter referred to as target genes) and model therapeutic glycoproteins (hereafter referred to as model proteins) to express. Furthermore, clonal variation is not an issue when a pool of transfected cells are analysed^6^. Generic antibody-independent product titer determination methods would facilitate complete interchangeability of model proteins because specific antibodies for each individual model protein would not be needed. Split-GFP complementation-based product titer assays provide such an antibody-independent solution^11^.

Recent advances in the split-GFP complementation technique have enabled a relatively small part (16 amino acid residues) of GFP to be tagged to the protein of interest; which is the 11^th^ β-strand of GFP’s β-barrel (S11)^11^. The remaining part of GFP (GFP1–10; 214 amino acid residues) has been optimized for complementation and solubility (GFP1–10_OPT_)^11^. Separately, S11 and GFP1–10_OPT_ are principally non-fluorescent; however, when the two fragments are mixed, the chromophore of GFP is formed and fluorescence complementation takes place^12^. Fluorescence intensity upon complementation is proportional to the concentration of the S11-tagged protein when GFP1–10_OPT_ is in excess^11^, making this technique useful for product titer determination. A variant of the S11 peptide (M3 variant; S11_M3) was selected based on good complementation characteristics and minimal perturbation on solubility of tagged proteins in *E. coli*^11^. The S11_M3-tag is therefore less likely to perturb folding processes and solubility than previous, larger versions of the split-GFP tag^13^,^14^.

In the present study, we chose two non-mAbs as model proteins: human α_1_-antitrypsin (hα1AT) and human C1 esterase inhibitor (hC1INH). hα1AT is a protein of 394 amino acid residues with three *N*-glycans^15^; hC1INH (478 amino acid residues) contains six *N*-glycans and at least seven *O*-glycans^16^. Currently, hα1AT and hC1INH are not produced commercially in cultured mammalian cells but are derived from human plasma for therapeutic use^15^,^17^. In addition, recombinant hC1INH (Ruconest^TM^) produced in milk of transgenic rabbits has recently been approved as a biopharmaceutical product^18^.

We set out to establish a miniaturized and parallelisable transient transfection-based screening platform to improve the productivity of therapeutic glycoproteins in CHO cells (Fig. 1). The platform combines four techniques suitable for the 96-well format: (1) Lipid-based transfection and transient overexpression (2) Cell cultivation in half-deepwell (HDW) microplates. (3) Fluorescence image cytometry-based cell counting. (4) Split-GFP-based product titer assay. We wanted to investigate the general applicability and versatility of the platform. To this end, we analysed the effect on productivity of hα1AT and hC1INH of overexpressing previously published target genes in CHO cells. By using this screening platform, we were able to demonstrate gene-specific increase in titer and *q*_p_ for our two model proteins.

## Materials and Methods

### Plasmids and primers

All primers used in this study are listed in Supplementary Table S1. The elements inserted into all constructs were verified by sequencing, and purified plasmid was obtained using the Nucleobond® Xtra Midi Kit (Machery-Nagel, Düren, Germany) according to the manufacturer’s instructions.

Plasmids encoding Rituximab and codon-optimized human Erythropoietin (hEPO) have previously been described^19^. The human genes *Serpina1* and *Serping1*, which encode hα1AT and hC1INH, respectively, either untagged or tagged with S11_M3 or S11_H7 (H7 variant of S11), were subcloned into pcDNA3.1(+)/Zeo (for schematic overview see Supplementary Fig. S1a; for sequences, see Supplementary Table S2).

Solid-phase cloning (SPC)^20^ was used to fuse the S11_M3-tag to the C-terminus of hC1INH. pcDNA3.1(+)/Zeo encoding hC1INH (PL_hC1INH_notag; Supplementary Table S3) was PCR amplified using Phusion® Hot-Start Flex (New England Biolabs, Ipswich, Massachusetts, USA) and primers #131 and #132. Primer #133 was designed to include 30 bases for hybridization, an amino acid linker (GGGSGGGS), and the S11_M3 sequence. The oligo (primer #133) was genetically coupled to the C-terminus of *Serping1* by head-to-tail SPC on a Magnatrix 1200 workstation (NorDiag AS, Oslo, Norway).

The remaining hα1AT and hC1INH constructs (Supplementary Table S4) were cloned by seamless PCR-based uracil-specific excision reagent (USER) fusion cloning, essentially as previously described^21^, using primers designed by the AMUSER software^22^. In brief, PCR amplicons (Supplementary Table S5) were made using the proofreading polymerase *Pfu*X7^23^ and assembled by treating with USER Enzyme (New England Biolabs) and transforming into *E. coli* One Shot® Mach1™ competent cells (Life Technologies, Thermo Scientific, Rockford, IL).

All target genes were cloned into a vector harbouring an ‘Nt.BbvCI/PacI A’ USER cassette (PL_TGExpr) (Supplementary Fig. S1b). PL_TGExpr was made using Flexible Assembly Sequence Tag-USER-based cloning (FAST-USER) as previously described^24^ (Supplementary Table S6). Coding sequences of target genes were PCR-amplified using *Pfu*X7 polymerase and primers designed by AMUSER software (Supplementary Table S5). Coding sequences were inserted into the ‘Nt.BbvCI/PacI A’ USER cassette of PL_TGExpr as previously described^25^ (Supplementary Table S6).

### Cell cultivation

CHO-S suspension cells (Life Technologies) were grown in CD CHO medium supplemented with 8 mM L-glutamine and 2 μL/mL anti-clumping agent (Life Technologies). Cells were expanded in Corning vent cap shake flasks (Sigma-Aldrich, St. Louis, MO) in a humidified incubator at 120 rpm (25 mm orbit), 37 °C, and 5% CO_2_.

### Transfection

Transfection in shake flasks, 6-well plates and 96-well microplates is described in Supplementary Information.

### 96-well based cell counting assay

Dye master mix was made daily: CD CHO + 8 mM L-glutamine + 5 μg/mL Hoechst-33342 (Life Technologies) + 0.4 μg/mL propidium iodide (Life Technologies). Master mix (200 μL) and cell suspension (3 μL) were mixed in a 96-well optical-bottom microplate (Greiner Bio-One, Frickenhausen, Germany) and cells were incubated for 40 min at RT. Image cytometry analysis was performed on a Celigo Imaging Cell Cytometer (Nexcelom Bioscience, MA, USA). Cells were identified using the blue channel (Hoechst-positive cells), and the red fluorescence channel (propidium iodide) was used to detect dead cells. Total cell density was computed from the total number of identified cells in the well. Cell viability was defined as the percentage of propidium iodide-negative cells. Based on total cell density and viability, viable cell density (VCD) was determined.

For comparison, VCD measurements were performed on a NucleoCounter NC-200 Cell Counter (ChemoMetec, Allerod, Denmark) using Via1-Cassettes™ using a Viability and Cell Count Assay (NucleoView software ver. 1.1.18.1) according to the manufacturer’s instructions.

### Deglycosylation of proteins

*N*-glycans on proteins in supernatant samples were removed by PNGase F treatment (New England Biolab) under denaturing conditions according to the manufacturer’s instructions. *N*-glycans and *O*-glycans on proteins in supernatant samples were removed using Protein Deglycosylation Mix (New England Biolab) under denaturing conditions according to the manufacturer’s instructions.

### Western blot analysis

All samples for Western blotting were subjected to reducing SDS-PAGE on NuPAGE® Novex® 4–12% Bis-Tris Protein Gels (Life Technologies) in MOPS running buffer before transfer onto nitrocellulose membrane using the iBlot® 2 Dry Blotting System (Life Technologies). Primary antibodies: polyclonal goat hα1AT antibody (#ab7633, Abcam, Cambridge, UK) and rabbit polyclonal hC1INH antibody (#ab97348, Abcam). Secondary antibodies: horseradish peroxidase (HRP)-conjugated polyclonal donkey anti-goat IgG secondary antibody (#31458, Pierce, Thermo Scientific) and HRP-conjugated polyclonal goat anti-rabbit IgG secondary antibody (#ab6741, Abcam). Bound antibody was detected with ECL Prime Western Blotting Detection Reagent (GE Healthcare, Little Chalfont, United Kingdom).

### Split-GFP product titer assay

Split-GFP product titer assay was performed essentially as described elsewhere^26^, but with minor changes to the protocol (see Supplementary Information).

### Protein quantification by ELISA and bio-layer interferometry

The hα1AT titer in supernatant samples was determined using a hα1AT ELISA Kit (#OKIA00048; Aviva Systems Biology, San Diego, CA, USA) according to the manufacturer’s instructions. The hα1AT calibrator from the kit (lyophilized serum) was used as standard. The titer of hC1INH in supernatant samples was determined using a hC1INH ELISA pair set (#SEK10995; Sino Biological Inc., Beijing, China) according to the manufacturer’s instructions. Purified recombinant his-tagged hC1INH (#10995-H08H, Sino Biological) was used as standard. The titer of hEPO in supernatant samples was determined using a hEPO ELISA Kit (#ab119522, Abcam) according to the manufacturer’s instructions. Purified recombinant hEPO (#Z02975-50, GenScript, Piscataway, NJ, USA) was used as standard. The Rituximab titer in supernatant samples was determined by bio-layer interferometry using an Octet RED96 (ForteBio, Pall, Menlo Park, CA, USA) as previously described^27^, but with an increased shaking speed of 1000 rpm.

### Calculation of productivity parameters

Integral of viable cell density (IVCD) and daily specific production rate were calculated as described elsewhere^6^.

### Statistical analysis

Data (fold-changes) were tested for normality by D’Augustino-Pearson omnibus test using GraphPad Prism software (version 6.05 for Windows, GraphPad Software, La Jolla, CA, USA). Data (non-transformed and log2-transformed) in some groups did not pass the normality test, so statistical significance (p ≤ 0.05) was assessed by performing the Mann-Whitney nonparametric test (two-tailed) on non-transformed fold-changes using GraphPad Prism software.

## Results

### Establishment of a 96-well-based cell-counting assay

**Figure 1:**
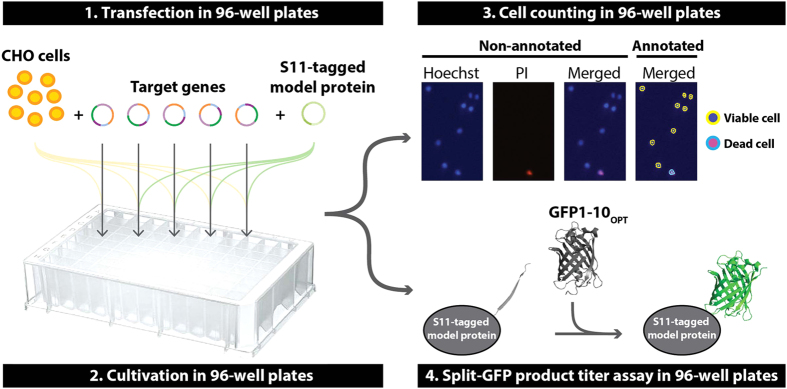
Schematic illustration of the screening platform. (**1**) CHO cells are co-transfected with plasmids encoding target genes and plasmids encoding S11(Split-GFP)-tagged model protein. (**2**) Transfected cells are cultivated in a 96-half-deepwell microplate capped with a low-evaporation Duetz sandwich cover (cover not shown). (**3**) Viable cell density is determined using image fluorescence cytometry on Hoechst and propidium iodide (PI) stained cells. (**4**) Upon addition of GFP1–10_OPT_ to supernatants, relative product titers of supernatants are determined by split-GFP complementation.

In order to monitor cell growth in 96-well microplates, we wanted to use a 96-well-based cell-counting assay compatible with small sample volumes. Based on a study by He and co-workers^28^, we established an assay that combines viability stain and 96-well-based imaging cell cytometry using 3 μL cell culture per sample (Fig. 1). Hoechst stain was used to identify the total number of cells (dead and alive) and propidium iodide stain was used to identify dead cells.

**Figure 2:**
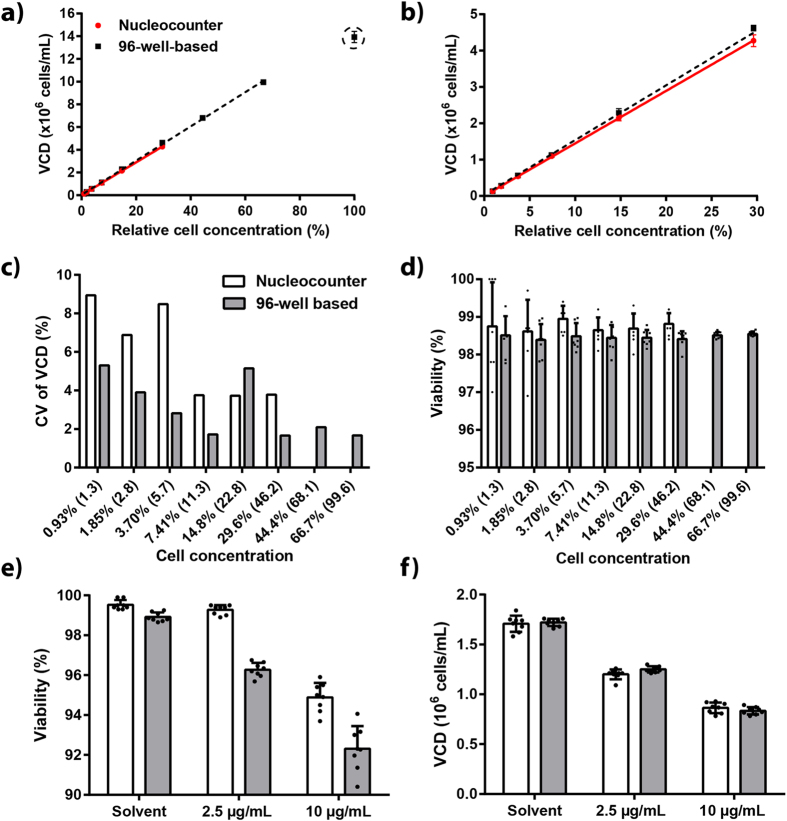
Wide linear range and low variation in 96-well-based cell counting assay. Comparison of NucleoCounter® NC-200 and 96-well-based (combined Hoechst and propidium iodide stain) cell-counting assay (eight technical replicates). (**a–d**) A dilution series of exponentially growing healthy CHO-S cells was analyzed. (**a**) Viable cell density (VCD) is plotted against relative cell concentration. Black rectangles and red circles denote data points from 96-well-based cell-counting and NucleoCounter assay, respectively. Mean and standard deviation are depicted. The dashed black line and solid red line show trend lines from linear regression of 96-well-based cell-counting (R^2^ = 0.999) and the NucleoCounter assay (R^2^ = 0.998), respectively. The encircled data point was found to be an outlier in the linear regression analysis. (**b**) Zoomed view of the graph shown in panel (**a**). (**c**) Coefficient of variation (CV) of VCD measurements at different cell concentrations. The x-axis denotes relative cell concentration and VCD values in brackets (×10^5^ cells/mL). White and grey bars denote the NucleoCounter and 96-well-based cell-counting assay, respectively. (**d**) Viability at different cell concentrations. The x-axis is as described in panel (**c**). Mean and standard deviation are depicted. (**e**–**f**) VCD and viability measurements of cells treated either with solvent (0.1% v/v DMSO) or with tunicamycin (2.5 or 10 μg/mL) for 24 h. White and grey bars denote the NucleoCounter and 96-well-based cell-counting assay, respectively. Mean and standard deviation are depicted.

Overall, VCD measurements of the 96-well-based assay were consistent with results obtained with the commercially available acridine orange- and DAPI-based NucleoCounter NC-200 Cell Counter (Fig. 2a,b). For analysis by the NucleoCounter NC-200 Cell Counter, dilution of cell cultures in which VCD exceeds 5 × 10^6^ cells/mL is recommended. The observed dynamic linear ranges of the NucleoCounter NC-200 Cell Counter and the 96-well-based cell-counting assay were 1 × 10^5^–5 × 10^6^ and 1 × 10^5^–1 × 10^7^ viable cells/mL, respectively (Fig. 2a–c). Intra-assay variation of VCD measurements was generally lower in the 96-well-based assay than in the NucleoCounter assay (Fig. 2c). Moreover, viability measurements were consistent between the two assays (Fig. 2d).

Next, we wanted to compare the performance of the two cell-counting assays with stressed cells. To this end, we treated CHO-S cells with the endoplasmic reticulum (ER)-stress inducer tunicamycin at two different concentrations for 24 h, because induction of ER stress affects the viability of mammalian cells^29^. Although lower viability values of the tunicamycin-treated cells were obtained in the 96-well-based assay compared with the NucleoCounter assay (Fig. 2e), VCD measurements of the two assays were consistent (Fig. 2f). Overall, the performance of the 96-well-based cell-counting assay was satisfactory and we concluded that it was suitable for monitoring cell growth in the 96-well format.

### Similar cell growth profiles and volumetric productivity in half-deepwells and shake flasks

**Figure 3:**
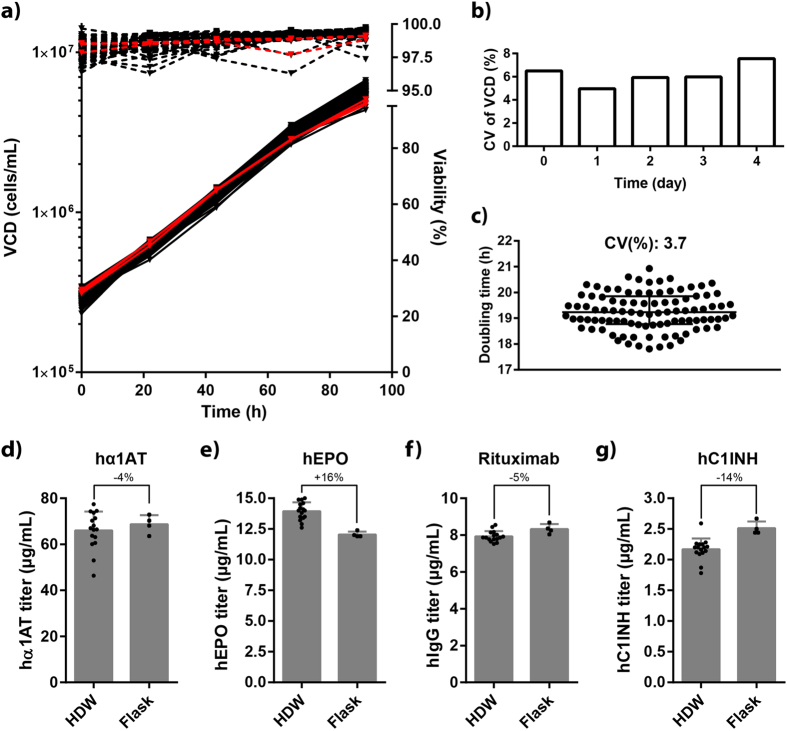
Shake-flask-like cell growth profile and volumetric productivity in a 96-HDW-microplate. (**a**–**c**) Comparison of cell growth in shake flasks (three flasks) and HDW-microplate (96 wells). CHO-S cells were seeded at 3 × 10^5^ cells/mL in shake flasks (25 mL in 125 mL flasks) or in HDWs (250 μL) and VCD and viability were measured daily (day 0–4). (**a**) VCD (solid lines) and viability (dashed lines) versus culture time. Red and black lines show data from shake flasks and HDWs, respectively. VCD values are plotted on a logarithmic scale. (**b**) Coefficient of variation (CV) of VCD measurements in HDWs during the four days of culture. (**c**) Doubling time from day 0–3 in 96 wells calculated by exponential growth equation (least squares fit) in GraphPad Prism (GraphPad Software). Median and interquartile range are depicted and CV is shown above the data points. (**d**–**g**) Comparison of volumetric productivity in shake flasks and HDWs of hα1AT, hEPO, Rituximab and hC1INH. CHO-S cells were transiently transfected with plasmids encoding the aforementioned proteins and supernatants two days post-transfection were obtained and titers were determined by ELISA (hα1AT, hEPO and hC1INH) and bio-layer interferometry (Rituximab). 16 and 4 separate transfections were performed in HDWs and flasks, respectively. Mean, standard deviation, and percentage difference are shown.

Having established a 96-well-based cell-counting assay, we were able to analyse cell growth in all wells in a 96-HDW-microplate and compare cell growth with standard Erlenmeyer shake flasks. Cells were seeded at 3 × 10^5^ viable cells/mL and VCD and viability were measured daily for four days. Cell growth profiles in shake flasks and in wells in a 96-HDW-microplate were comparable (Fig. 3a). Viability during the four days of culture was in general high (>95%) and was consistent between shake flasks and HDWs (Fig. 3a).

Variation of VCD measurements was in general low (coefficient of variation [CV] = 5–8%) during the four-day period (Fig. 3b). Therefore, the observed variation seems to originate from small differences in seeding density (Fig. 3b) and variation of the 96-well-based cell-counting assay (Fig. 2c). More importantly, the CV of doubling time was 3.7% from day 0–3 (Fig. 3c) demonstrating low well-to-well variation. Moreover, well position had no observed effect on cell growth rate (Supplementary Tables S7 and S8).

Next, we wanted to compare volumetric productivity of different therapeutic glycoproteins in HDWs and shake flasks. To this end, we chose our two model proteins hα1AT and hC1INH as well as hEPO^30^ and Rituximab^31^. Two days after transfection, supernatant samples were obtained and product titer was determined. In general, volumetric productivity of each of the four proteins was comparable in HDWs and shake flasks (Fig. 3d–g). Titer of hα1AT, Rituximab and hC1INH was on average 4%, 5% and 14% lower, respectively, in HDWs compared to shake flasks, whereas hEPO was 16% higher. Because we were able to demonstrate comparable cell growth profiles and volumetric productivity between shake flasks and HDWs, we inferred that the 96-HDW-microplate was a suitable small-scale cultivation vessel for use in our transient transfection-based screening platform.

### Comparison of S11 variants fused N- and C-terminally to model proteins

We wanted to investigate the applicability of using split-GFP methodology to determine product titer because this is a potentially versatile alternative to antibody-based product titer assays for non-mAbs (see Discussion). To this end, we tagged our two non-mAb model proteins (hα1AT and hC1INH) N- and C-terminally with two different variants of the split-GFP S11-tag: M3 and H7 (Supplementary Fig. S1a). The H7 variant has been shown to improve expression levels of membrane proteins in *E. coli* compared with the M3 variant^32^. The H7 variant (17 residues) is one amino acid residue longer than the M3 variant and contains seven histidine residues in contrast to the two histidine residues of M3 (Supplementary Fig. S2).

**Figure 4:**
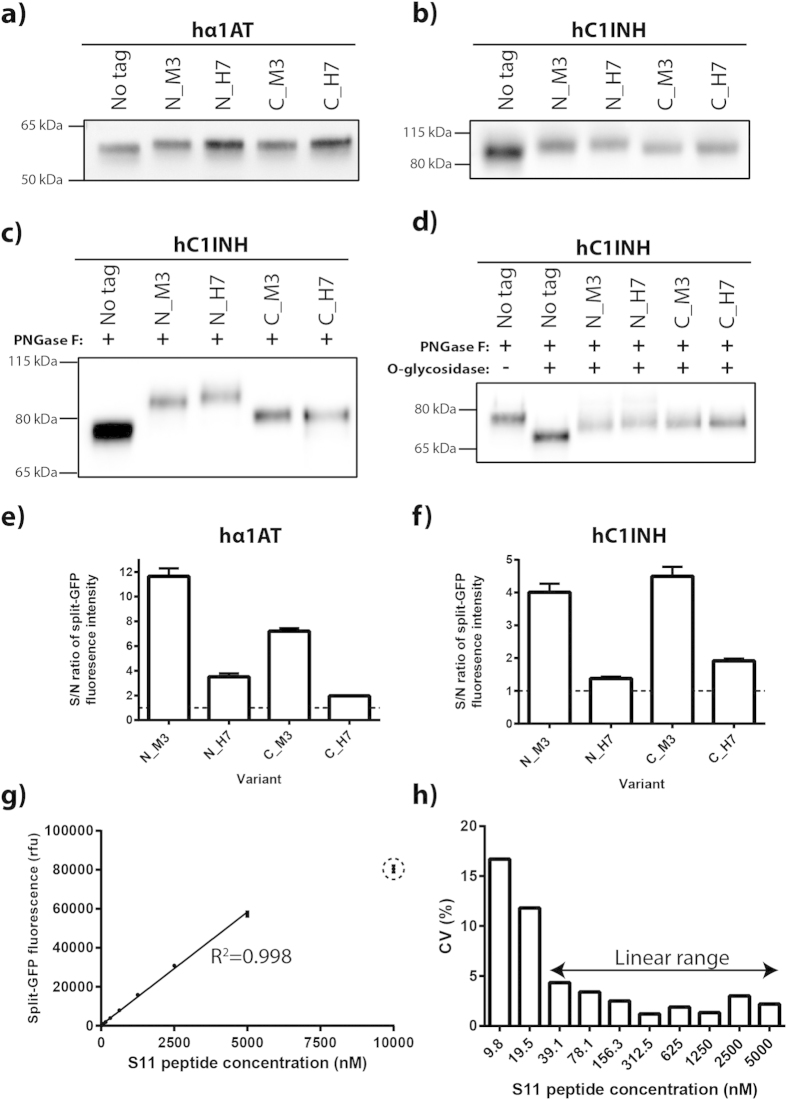
Analysis of split-GFP S11-tag variants. (**a**–**f**) CHO-S cells in 6-well plates were transfected with plasmids encoding hα1AT or hC1INH either S11_M3 or S11_H7-tagged at the N- or C-terminus or untagged. Supernatants two days post-transfection were analyzed. (**a**) α-hα1AT and (**b**–**d**) α-hC1INH Western blots under reducing conditions. The Western blots shown are representative of three experiments. (**c**,**d**) Proteins in supernatants were subjected to PNGase F and *O*-glycosidase (Protein Deglycosylation Mix) treatment under denaturing conditions where indicated. (**e**,**f**) Supernatants were analyzed by split-GFP product titer assay. Signal-to-noise (S/N) ratio was determined by defining noise as split-GFP complementation signal from mock transfected cells. Mean and standard deviation are depicted (n = 3). (**g**) A dilution series of a synthetic S11_M3 peptide was analyzed by split-GFP product titer assay. The trend line from linear regression and correlation coefficient (R^2^) are shown. The encircled data point was found to be an outlier in the linear regression analysis. Mean and standard deviation from eight technical replicates are depicted. (**h**) Intra-assay variation of the split-GFP product titer assay was analyzed from the data shown in panel (**e**). The coefficient of variation (CV) at different concentrations of the S11_M3 peptide are shown. The two-headed arrow denotes the defined dynamic linear range with low intra-assay variation (CV < 10%).

All hα1AT and hC1INH variants were expressed transiently for two days. The H7 variant seemed to increase the titer of hα1AT, whereas productivity of hα1AT appeared unaffected by the M3 variant (Fig. 4a). In contrast, both variants gave rise to decreased titer of hC1INH (Fig. 4b) indicating that the effects of S11-fusion are protein-specific. C-terminally tagged hC1INH variants migrated slightly slower than untagged hC1INH consistent with fusion of the S11-tag, which is approximately 2 kDa in size (Fig. 4b). However, the N-terminally tagged hC1INH variants reproducibly migrated even slower than the C-terminally tagged hC1INH variants (Fig. 4b). This gel migration pattern was retained upon removal of *N*-glycans by PNGase F (Fig. 4c). However, removal of *N*- and *O*-glycans gave rise to co-migration of the N-terminally tagged M3 variant with the C-terminally tagged variants (Fig. 4d). Removal of *N*- and *O*-glycans also gave rise to a notable migration shift for the N-terminally tagged H7 variant but this variant reproducibly migrated slightly slower than the other three S11-tagged variants. The glycosidase mix used in this analysis is not able to remove all possible *O*-glycan structures. Thus, the N-terminally tagged H7 variant might contain such resistant *O*-glycan structure(s). We concluded that *O*-glycosylation was affected when the S11 peptide was N-terminally fused to hC1INH. Apart from this, all S11-tagged variants of hα1AT and the C-terminally tagged variants of hC1INH appeared as expected; migrating slightly slower than the untagged variants consistent with fusion of the S11-tag (Fig. 4a,b).

Preliminary results indicated that the H7 variant gave rise to lower fluorescence complementation intensity than the M3 variant upon split-GFP complementation (data not shown). In order to elucidate this, we compared signal-to-noise ratios of all S11 constructs. All M3 variants gave rise to higher signal-to-noise ratio than the H7 variants (Fig. 4e,f), despite their titers being similar or lower (Fig. 4a,b). This suggests that the M3 variants have higher complementation capacity: thus, we chose this S11-tag variant for further experiments. We chose N-terminally tagged hα1AT and C-terminally tagged hC1INH for all further experiments.

To determine the dynamic linear range of the split-GFP assay, we subjected a dilution series of a synthetic S11_M3 peptide to split-GFP product titer assay. Consistent with previously reported data^11^, we observed a clear linear relationship between split-GFP complementation intensity and peptide concentration (Fig. 4g). The dynamic linear range spanned two orders of magnitude and showed intra-assay variation below 10% (Fig. 4h). This suggests that substantial changes in titer can be quantified without additional dilution steps.

### Split-GFP-based overexpression platform reveals target gene-specific effects on productivity

Having all required techniques established, we wanted to test the applicability of our screening platform. As proof of concept, we tested seven target genes encoding proteins with previously reported effects on the productivity of recombinant glycoproteins in CHO cells (for reviews see^7^,^9^): five genes with positive effects (*Xbp1s, Pdia3, Ppp1r15a, Ero1l,* and *Atf4*) and two genes with mixed effects (*Hspa5* and *P4hb)*. Although not previously tested, *Hsp90b1* was also included because its expression has been found to positively correlate with heterologous protein production^33^. The functions of the proteins encoded by these eight genes are all related to the ER, as they are either involved in the unfolded protein response or are chaperones and/or oxidoreductases localized in the ER. As negative controls, two pro-apoptotic genes were included: *Bak1* and *Bax*^34^.

Eight individual transfection pre-mixes were made per target gene because we observed some transfection-based variation (Supplementary Fig. S3). Although not separated in time, data from individual transfections were processed statistically as individual data points in order to cover as much variation as possible.

**Figure 5:**
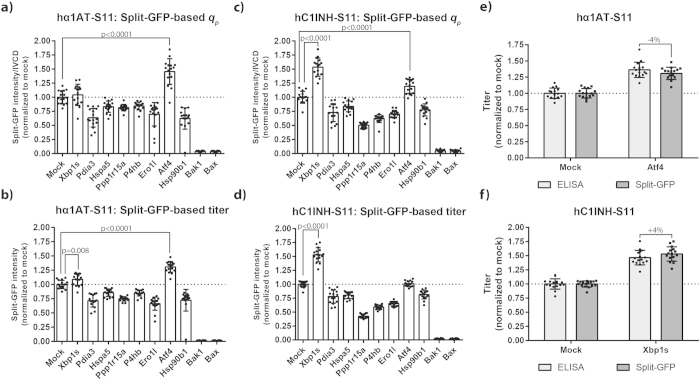
Co-expression of target genes affects split-GFP-based specific and volumetric productivity of hα1AT-S11 and hC1INH-S11. (**a**,**b**) Plasmid encoding hα1AT N-terminally tagged with S11_M3 was co-transfected with mock plasmid or plasmids encoding target (mouse) genes into CHO-S cells in 96-HDW-microplates. Three days after transfection, supernatants were obtained and subjected to split-GFP product titer assay for determination of relative titer. Split-GFP-based specific productivity (*q*_*p*_) and titer are shown. Eight separate transfections were performed in each experiment and experiments were performed twice (n = 2; 16 wells). Mean and standard deviation are depicted. Only statistically significant (p ≤ 0.05) increases in productivity are shown. (**c**,**d**) As described for panels (**a,b**), with the only difference being use of plasmid encoding hC1INH C-terminally tagged with S11_M3 instead of plasmid encoding hα1AT. (**e**,**f**) Comparison of split-GFP-based product titer assay and ELISA. hα1AT (mock and *Atf4* ) and hC1INH (Mock and *Xbp1s*) supernatants described in panels a-d were subjected to ELISA for determination of hα1AT and hC1INH titers. Mean, standard deviation and percentage difference are shown.

Co-expression of *Atf4* gave rise to increased *q*_p_ and titer of hα1AT-S11 three days post-transfection (Fig. 5a,b). As for hC1INH-S11, co-expression of *Xbp1s* gave rise to increased *q*_p_ and titer (Fig. 5c,d). Although less prominent, effects of *Atf4* and *Xbp1s* were also observed two days post-transfection (Supplementary Fig. S4). Interestingly, three of the five genes previously reported to have a positive effect on specific productivity had a negative effect on both model proteins (Fig. 5a–d): *Pdia3*, *Ppp1r15a,* and *Ero1l*. Apart from reduced viability for *Ppp1r15a*, only slight differences in viability (Supplementary Fig. S5) and IVCD (Supplementary Fig. S6) were observed. Minute amounts of hα1AT-S11 and hC1INH-S11 were produced upon co-expression of *Bax* and *Bak1* (Fig. 5a–d). As expected from these pro-apoptotic genes, a significant drop in viability was observed (Supplementary Fig. S5) resulting in a drop in IVCD (Supplementary Fig. S6).

In order to validate the split-GFP product titer assay for our two model proteins, we wanted to compare this assay with the widely used protein quantification method; ELISA. To this end, product titer was determined in selected hα1AT and hC1INH supernatant samples by ELISA (Fig. 5e,f). Relative titer determined by split-GFP complementation for hα1AT-S11 and hC1INH-S11 was within ±4% compared to ELISA. Thus, we concluded that the split-GFP product assay is able to accurately quantify relative titers of therapeutic glycoproteins in complex samples (spent media).

### Positive effects of *Atf4* and *Xbp1s* validated on untagged model proteins

**Figure 6:**
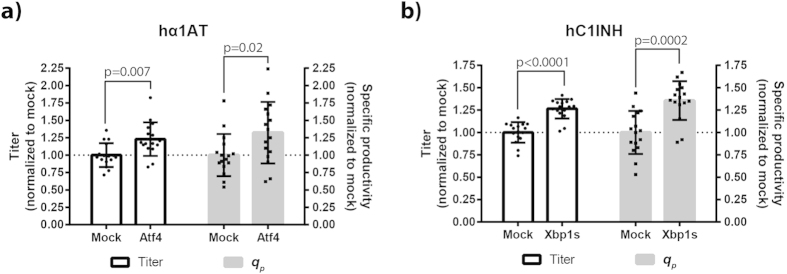
Positive effects of target genes on productivity validated on untagged hα1AT and hC1INH. (**a**) Plasmid encoding untagged hα1AT was co-transfected with a mock plasmid or plasmid encoding *Atf4* into CHO-S cells in a 96-HDW-microplate. Three days after transfection, supernatants were obtained and hα1AT titer determined by ELISA. Normalized titer and specific productivity (*q*_*p*_) are shown. Eight separate transfections were performed in each experiment and experiments were performed twice (n = 2; 16 wells). Mean, standard deviation, and statistically significant (p ≤ 0.05) fold-changes are depicted. (**b**) Plasmid encoding untagged hC1INH was co-transfected with mock plasmid or plasmid encoding *Xbp1s* into CHO-S cells in 96-HDW-microplate. Three days after transfection, supernatants were obtained and hC1INH titer determined by ELISA. Normalized titer and specific productivity (*q*_*p*_) are shown. Eight separate transfections were performed in each experiment and experiments were performed twice (n = 2; 16 wells). Mean, standard deviation, and statistically significant (p ≤ 0.05) fold-changes are depicted.

Lastly, we wanted to test whether the positive effects of *Atf4* and *Xbp1s* could be detected on untagged hα1AT and hC1INH. To this end, untagged hα1AT and hC1INH were expressed in CHO-S cells in a 96-HDW-microplate for three days with and without co-expression of *Atf4* and *Xbp1s*. Titer and *q*_p_ for untagged hα1AT and hC1INH co-expressed with a mock plasmid were 72 mg/L and 13 pcd, and 3.2 mg/L and 0.5 pcd, respectively. Increased titer and *q*_p_ were observed for hα1AT and hC1INH upon co-expression of *Atf4* and *Xbp1s*, respectively (Fig. 6a,b). Although the effects were 8–16% higher with S11-tagged than untagged model proteins (Supplementary Table S9), we concluded that our split-GFP-based transient expression platform is able to identify effects on productivity of non-mAb glycoproteins.

## Discussion

Compared with conventional shake flask cultivation of CHO cells, cultivation in 96-well microplates significantly increases throughput. Originally, growth conditions in 96-well microplates were suboptimal due, for example, to excessive evaporation and insufficient oxygen transfer rate^35^. However, these issues have been solved by Duetz covers^36^, which reduce evaporation while allowing sufficient oxygen transfer. The dramatic effect of the Duetz cover on evaporation was recently demonstrated by showing a 5.5-fold decrease in evaporation compared with gas-permeable seals^37^. In the present study, evaporation of 1.4% v/v per well per day was observed in 96-HDW-microplates with Duetz covers (data not shown). We were able to demonstrate cell-growth profiles in 96-well microplates that were comparable to those in shake flasks (Fig. 3a). Similarly, shake-flask-like cell-growth profiles have been reported in 24-well plates using Duetz covers^38^. The well-to-well variation of VCD on day 4 in 24-well plates was reported to be 9.0%^38^, which is comparable with the value of 7.5% observed in 96-well microplates in this study (Fig. 3b). No effect of well position on cell growth rate was observed in 24-well plates, which is consistent with our results in 96-well microplates (Supplementary Table S7).

Similarly, we observed relatively low variation of titer and *q*_p_ (Figs 5 and 6). Mean variation of titer and *q*_p_ was 9.7% and 14.9%, respectively, for both model proteins and all target genes except *Bak1* and *Bax* (data from Fig. 5). The variation of *q*_p_ is consistent with the previously reported variation of *q*_*antibody*_ (16%) from polyethylenimine-mediated transfection of CHO cells in flasks^10^. The variation of titer is comparable with the observed variation (CV: 9.2%) in transfection efficiency, which ranged from 55% to 80% (Supplementary Fig. S3). This range likely explains why unexpectedly low titers and *q*_p_ were observed for some wells (*e.g. Ero1l* and *Hsp90b1* in Fig. 5a,b and *Atf4* in Fig. 6a). To our knowledge, this is the first published example of transient protein production in CHO cells cultivated in 96-well microplates. Recently, a study was published on transient expression of IgG1 in HEK293 cells by polyethylenimine-mediated transfection in 96-deepwell microplates using Duetz covers^37^. Unfortunately, comparison of well-to-well variation from that study is not possible, because only plate-to-plate variation was reported. Importantly, we were able to show that volumetric productivity of four different therapeutic glycoproteins in shake flasks was comparable in HDWs (Fig. 3d–g). However, some differences were observed ranging from −16% to +14% between shake flasks and HDWs. Moreover, variation in titer was higher in HDWs compared to shake flasks: average CV of all four proteins HDWs and shake flasks was 7% and 4%, respectively. Overall, it is likely that these differences partly arise from differences in the transfection procedure. Complexation of the transfection reagent and plasmids is a time-dependent process^39^ and the complex incubation time likely varies more when transfecting cells in HDWs compared to shake flasks. Automation of the transfection process by a liquid handling robot could potentially minimize this variation to some extent.

Sixteen wells per gene were used in the screening platform. The sample size can be reduced to three and six wells to detect fold-changes of 1.5 to 1.3, respectively, with power of 0.80 and type I error of 0.05 based on the observed variation of 15%. Moreover, several plates can be analysed in parallel. Finally, the screening platform is suitable for automation by a liquid handling robot because the majority of the experimental work involves liquid transfer between 96-well microplates. These options have the potential to increase the throughput and number of identified positive effects significantly of the screening platform. Importantly, positive effects on productivity of target genes must eventually be further characterized in an industry-like context as well as product quality attributes such as glycosylation and bioactivity.

Effects on productivity were less pronounced on untagged model proteins than S11-tagged model proteins (Supplementary Table S9). This discrepancy is likely a consequence of fusing the S11-tag to the model proteins. Although the S11-tag is relatively small (~2 kDa), fusion of this tag to proteins could change the overall conformation as well as post-translational modifications. Indeed, the H7 S11-variants of hα1AT led to increased titers and all S11-tagged variants of hC1INH gave rise to decreased titers compared to untagged protein (Fig. 4a,b). Moreover, N-terminally fusion of the S11 tag to hC1INH altered *O*-glycosylation (Fig. 4c,d). These results clearly show that fusion of the S11-tag to the protein of interest is a possible drawback of this platform. However, further optimization of the S11-tag sequence might be able to address this issue to some extent – for example by altering the glycine-serine linker sequence^40^. Despite the effects of the S11-tag on titers and post-translational modifications, we could reproduce that *Atf4* and *Xbp1s* overexpression significantly improved productivity of untagged hα1AT and hC1INH, respectively (Fig. 6a,b). Moreover, relative titers obtained by ELISA and split-GFP complementation were consistent (Fig. 5e,f). Thus, split-GFP complementation seems to be a versatile antibody-independent method for determining relative product titer of recombinant glycoproteins produced in CHO cells. For mAbs, several well-established high-throughput and automatable product titer assays exist. However, this is not the case for the majority of non-mAbs; split-GFP-based product titer determination therefore seems particularly valuable for non-mAb recombinant proteins. Besides the application as a discovery tool in the platform presented here, the split-GFP methodology could be useful in initial trials of producing protein variants by transient expression in the biopharmaceutical industry. However, the split-GFP system is not suitable for larger scale production in stable cell lines, because the S11-tag eventually becomes an undesired trait in for example detailed characterization of the protein and in clinical trials.

Five genes previously reported to have positive effects on productivity of recombinant glycoproteins in CHO cells were studied: *Atf4, Xbp1s*, *Pdia3, Ppp1r15a*, and *Ero1l. Atf4* and *Xbp1s* increased productivity in a product-specific manner (Fig. 5). It is unclear whether this is because of product specificity, due to a possible difference in baseline *q*_p_ of hα1AT and hC1INH, or a combination thereof. In contrast, *Pdia3, Ppp1r15a*, and *Ero1l* did not increase productivity in the present study (Fig. 5). There are several plausible reasons for this, which are discussed here: (1) *Effects of target genes are model protein-specific*. Owing to the interchangeable and parallelisable setup of the screening platform, model protein-specific effects can readily be studied by testing different model proteins (2) *Expression levels of target genes affect productivity.* Dose-dependence of target genes can be studied in a high-throughput manner by titrating the target gene-encoding plasmid with non-specific (filler) plasmid^6^,^41^,^42^. (3) *q*_*p*_ *may be too low in transient expression setups for rate-limiting steps to form in the secretory pathway similar to those observed in highly gene-amplified stable clones.* Relatively high transfection efficiency was obtained (Supplementary Fig. S3); consequently, substantial increase in *q*_p_ may be difficult to obtain by optimizing the current transfection setup. Alternatively, episomal replication-proficient CHO cell lines^43^,^44^ or stable CHO cell pools using the piggyBac transposon system^45^ could be used to increase and sustain productivity. (4) *Mouse-derived genes may not be functional in CHO cells*. Although mouse and hamster are closely related animals in an evolutionary context, proteins encoded from mouse genes might not be fully functional in CHO cells due to for example altered codon usage and post-translational modifications. Moreover, favourable interactions might not be compatible between a mouse gene-encoded protein and a human therapeutic glycoprotein in CHO cells. While not all mouse genes may be appropriate for expression in CHO cells, mouse genes have previously been used successfully to improve productivity of recombinant glycoproteins in CHO cells^46^,^47^. Furthermore, both pro-apoptotic genes (*Bak1* and *Bax*) clearly affected cell viability (Supplementary Fig. S5). Alternatively, mouse genes can be replaced by homologous hamster genes owing to the recent drafts of the Chinese hamster and CHO cell line genomes^48^,^49^,^50^,^51^. However, not all hamster genes are at present annotated correctly when compared to mouse homologues (our unpublished observations). Consequently, using hamster genes in our screening platform could lead to erroneous conclusions, until sequencing and annotation of the hamster genome have been further refined.

Combinatorial cell line engineering seems to be a constructive way of improving secretory capacity and several examples of synergistic effects on productivity of co-expressing two target genes have been reported^52^,^53^,^54^. More than 20,000 genes are present in the Chinese hamster genome^49^ and combinatorial approaches dramatically increase the number of conditions possible to be tested. Compared with current lower throughput setups, our platform facilitates cost-efficient combinatorial screening of genes. In conclusion, we believe that our target gene-screening platform holds the potential to unravel novel genes and combinations of genes that can enhance the secretory pathway machinery in CHO cells.

## Additional Information

**How to cite this article**: Hansen, H. G. *et al.* Versatile microscale screening platform for improving recombinant protein productivity in Chinese hamster ovary cells. *Sci. Rep.* **5**, 18016; doi: 10.1038/srep18016 (2015).

## Supplementary Material

Supplementary Information
