# Supplementary material for: Versatile microscale screening platform for improving recombinant protein productivity in Chinese hamster ovary cells
Source: Sci Rep. 2015 Dec 11;5:18016. doi: 10.1038/srep18016 (PMC4676018; doi:10.1038/srep18016)
Supplement: Supplementary Information [file srep18016-s1.pdf]

Supplementary information

Versatile microscale screening platform for improving recombinant protein productivity in Chinese hamster ovary cells

Henning Gram Hansen[1], Claes Nymand Nilsson[1], Anne Mathilde Lund[2], Stefan Kol[1], Lise Marie Grav[1], Magnus Lundqvist[3], Johan Rockberg[3], Gyun Min Lee[1,4], Mikael Rørdam Andersen[2] and Helene Faustrup Kildegaard[1,*].

1: The Novo Nordisk Foundation Center for Biosustainability, Technical University of Denmark (DTU), Hørsholm, Denmark.

2: Department of Systems Biology, Technical University of Denmark (DTU), Lyngby, Denmark.

3: Department of Proteomics and Nanobiotechnology, Royal Institute of Technology (KTH), Stockholm, Sweden.

4: Department of Biological Sciences, KAIST, Daejeon, Republic of Korea.

Supplementary information

Supplementary Materials and Methods

Transfection in 96-well microplates

Experiments were performed in pre-sterilized polystyrene 96-square System Duetz HDW-microplates (CR1496c, Enzyscreen, Haarlem, Netherlands) capped with autoclaved low-evaporation sandwich Duetz covers (CR1296a, Enzyscreen). Cells (250 µL culture per well) were incubated in a Multitron Cell humidified incubation shaker (Infors HT, Basel, Switzerland) at following conditions: 85% humidity, 37°C, 5% CO_2, 225 rpm (250 rpm when incubating transfected cells to minimize cell clumping), 50 mm orbit.

The day before transfection, anti-clumping agent was removed from cells maintained in shake flasks by two centrifugation-based (200*g*, room temperature [RT], 5 min) washing steps. Plasmids were diluted in OptiPRO™ SFM in a V-bottomed 96-well microplate (Greiner Bio-One). One half of total DNA amount was plasmid encoding model protein and the other half was either a mock plasmid (PL_TGExpr) or plasmids encoding target genes. Subsequently, FreeStyle™ MAX Reagent was diluted in OptiPRO™ SFM and immediately after mixed with diluted plasmid. After 5 minutes of complexation, plasmid:FreeStyle™ MAX reagent mix was transferred to wells in HDW-microplate containing 2.5×10^5 cells in 250 µL CD CHO supplemented with 8 mM L-Glutamine and 50 U/mL Penicillin-Streptomycin (Life Technologies). 3 hours post-transfection, anti-clumping agent was added to all wells reaching a final concentration of 2 µL/mL. VCD and viability were measured just before transfection (t=0) and at the following time points post-transfection: 24 h (day 1), 48 h (day 2) and 72 h (day 3). Supernatant samples were obtained by transferring cell suspension to V-bottomed 96-well microplate (Greiner Bio-One) and centrifuging (500*g*, RT, 5 min) the plate. Supernatants were recovered and stored at -80°C. When supernatant samples were obtained on day 2 and day 3 post-transfection (and not only on day 3), 30 µL cell suspension was diluted with 60 µL complete medium (3-fold dilution) to minimize effects of changing total cell culture volume.

Transfection in 6-well plates and shake flasks

Transfection was performed essentially as described for 96-well microplates. In brief, 3×10^6 cells (6-well plate) and 1×10^7 cells (shake flask) were transfected using FreeStyle™ MAX Reagent (Life Technologies) in 3 mL (6-well plate) and 10 mL (shake flask) CD CHO complete medium without anti-clumping agent according to manufacturer's instructions. Transfected cells were incubated in non-treated 6-well plates (Greiner Bio-One, Frickenhausen, Germany) and Corning vent cap shake flasks (Sigma-Aldrich) at 120 rpm (25 mm orbit), 37°C, and 5% CO_2. 3 hours post-transfection, anti-clumping agent was added to all wells reaching a 2 µL/mL final concentration.

Supplementary information

Transfection efficiency

Transfection efficiency was based on fluorescence of cells transfected with plasmid expressing enhanced YFP (PL_eYFP; Supplementary Table S3). Cells were Hoechst-stained as described for the 96-well based cell counting assay. Image cytometry analysis was performed on a Celigo Imaging Cell Cytometer with cells being identified as Hoechst-positive cells (blue fluorescence channel) and the green fluorescence channel was used to detect eYFP signal. Transfection efficiency was defined as the percentage of eYFP-positive cells.

Split-GFP product titer assay

Plasmid pGFPe_[1-10_{OPT}][1] was transformed into *E. coli* BL21 (DE3). Transformants were cultured in 500 mL 2xYT medium containing 50 µg/mL kanamycin until mid-exponential phase, at which time GFP1-10 expression was induced with 1 mM Isopropyl β-D-1-thiogalactopyranoside for 5 hours. After harvesting the cells at 4,000*g* for 10 min at 4°C, cells were lysed by homogenization on Emulsiflex-C5 (Avestin, Mannheim, Germany) in TNG buffer (100 mM Tris-HCl [pH 7.5], 150 mM NaCl, 10% glycerol vol/vol). Crude inclusion bodies were isolated by centrifugation at 30,000*g* for 30 min at 4°C and first washed three times with TNG buffer supplemented with 1 tablet/50 mL cOmplete EDTA-free protease inhibitor cocktail (Roche, Basel, Switzerland), 1% n-Dodecyl-β-D-Maltopyranoside (Anatrace, Maumee, OH, USA), 1 mg/mL lysozyme (Sigma-Aldrich), and 1 mM DTT (Sigma-Aldrich), and then twice with TNG buffer. After centrifugation at 16,000*g* for 10 min at RT, six aliquots of 1 mL of the pelleted inclusion bodies were stored at -80°C. To use GFP1-10_{OPT} for *in vitro* quantification, one aliquot was dissolved in 1 mL 9 M urea containing 5 mM DTT, centrifuged at 16,000*g* for 1 min at RT, diluted in 25 mL TNG buffer, and sterile filtered through a 0.2 µm syringe filter.

Quantification of GFP-fluorescence resulting from complementation between S11-tagged proteins and GFP1-10_{OPT} was performed essientially as previously described[2]. Briefly, purified GFP1-10_{OPT} was diluted 9-fold in TNG buffer. 20 µL supernatant samples were mixed with 180 µL diluted GFP1-10_{OPT} in black flat-bottomed 96-well microplates (Thermo Scientific). After overnight incubation in the dark at RT, fluorescence intensity was measured on a Synergy 2 Microplate Reader (BioTek Instruments Inc., Burlington, VT, USA) with 488 nm excitation and 510 nm emission with a 9 nm band pass (read height 8 mm) using same gain value for all plates. Background fluorescence of supernatant from mock-transfected cells (PL_pcDNA3.1/Zeo(+)) was subtracted for all samples.

A peptide with the amino acid sequence of the S11_M3 tag[3] was synthesized (GenScript, Piscataway, NJ, USA). Serial dilution of the S11_M3 peptide in complete CD CHO medium was performed to ensure linear relationship between split-GFP complementation signal and concentration of S11-tagged protein.

Supplementary information

Supplementary Figures

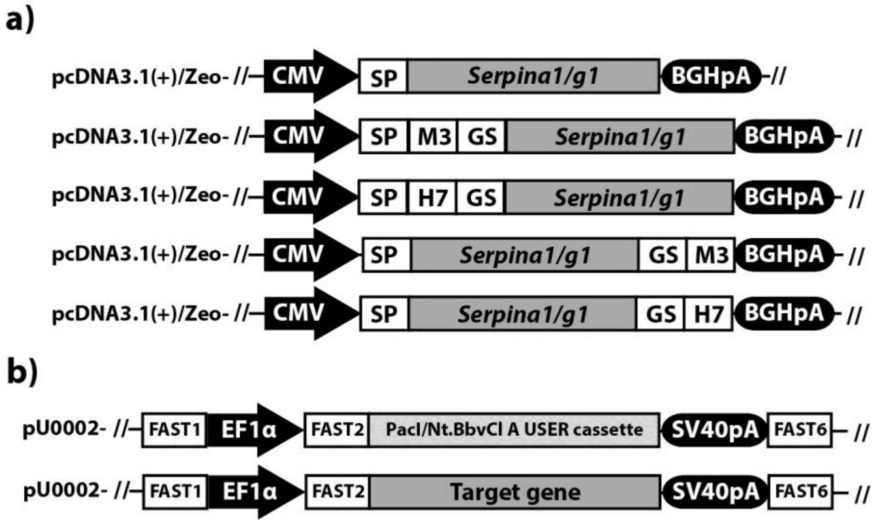

Figure S1. (a) Schematic representation of constructs expressing untagged or tagged hα1AT and hC1INH encoded by *Serpina1* and *Serping1* genes, respectively. SP: Signal peptide; M3: S11_M3 split-GFP tag; H7: S11_H7 split-GFP tag; GS: GS linker. (b) Schematic representation of pU0002-based vector[4] harboring EF1α promotor, PacI/Nt.BbvCI A USER cassette for insertion of target genes and SV40 polyadenylation signal (PL_TGExpr). Vector was assembled using Flexible Assembly Sequence Tags (FASTs)[5].

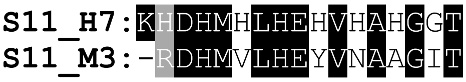

Figure S2. Alignment of S11_H7 and S11_M3 split-GFP peptides.

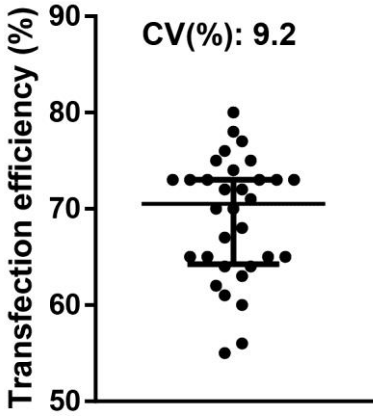

Figure S3. Variation of transfection efficiency in 96-HDW-microplates. CHO-S cells were transfected with plasmids encoding eYFP in 96-HDW-microplates and 32 separate transfections were performed (32 wells). Transfection efficiency was analyzed by fluorescence image cytometry 24 hours post-transfection. Median and interquartile range are depicted and CV is shown above the data points.

Supplementary information

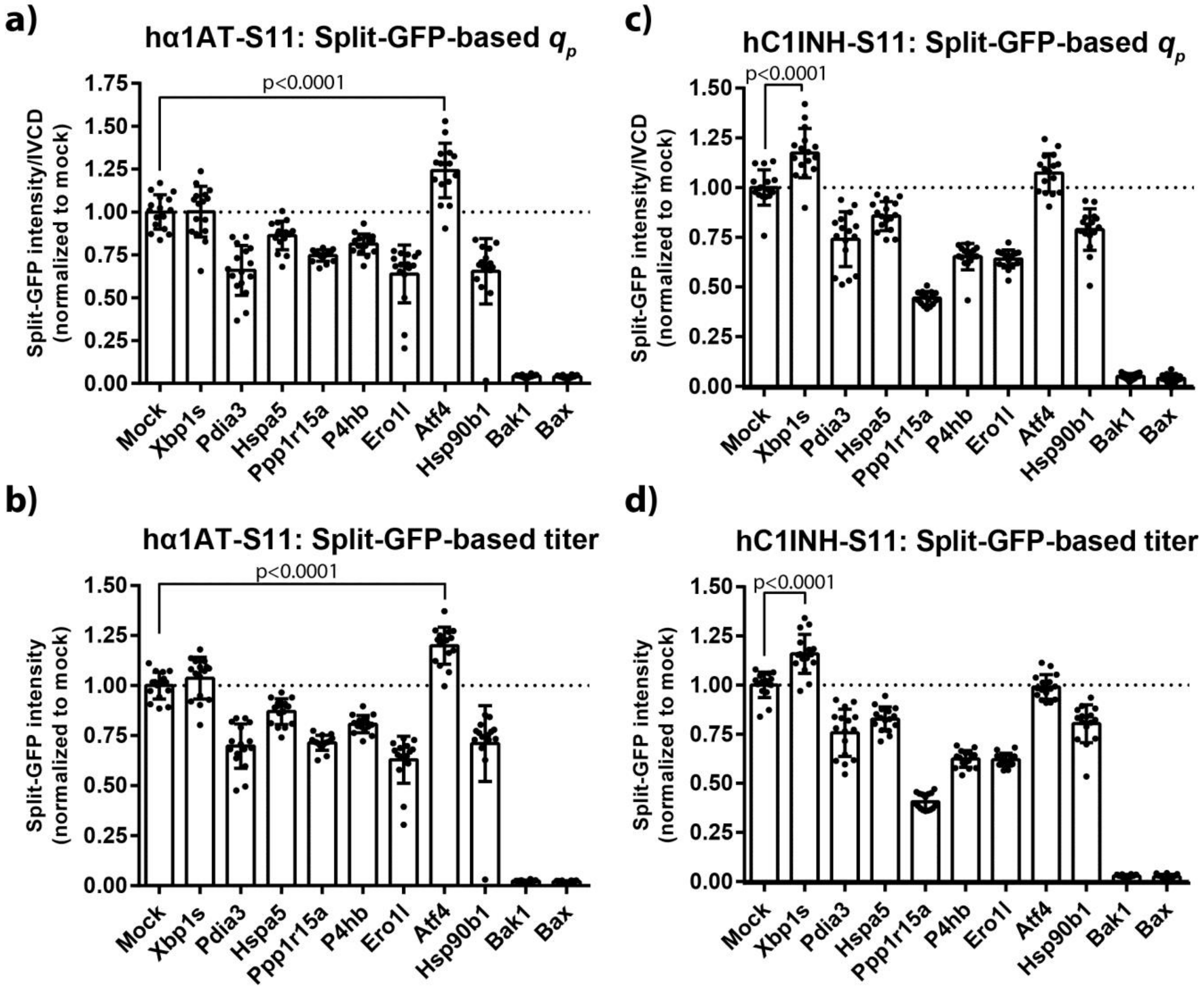

Figure S4. (a-d) Split-GFP-based specific productivity (q_p) and titer of cells co-transfected with plasmid encoding S11-tagged model protein and target genes **two days** post-transfection. Mean and standard deviation are depicted (n=2; 16 wells). See Figure 5's caption for details.

Supplementary information

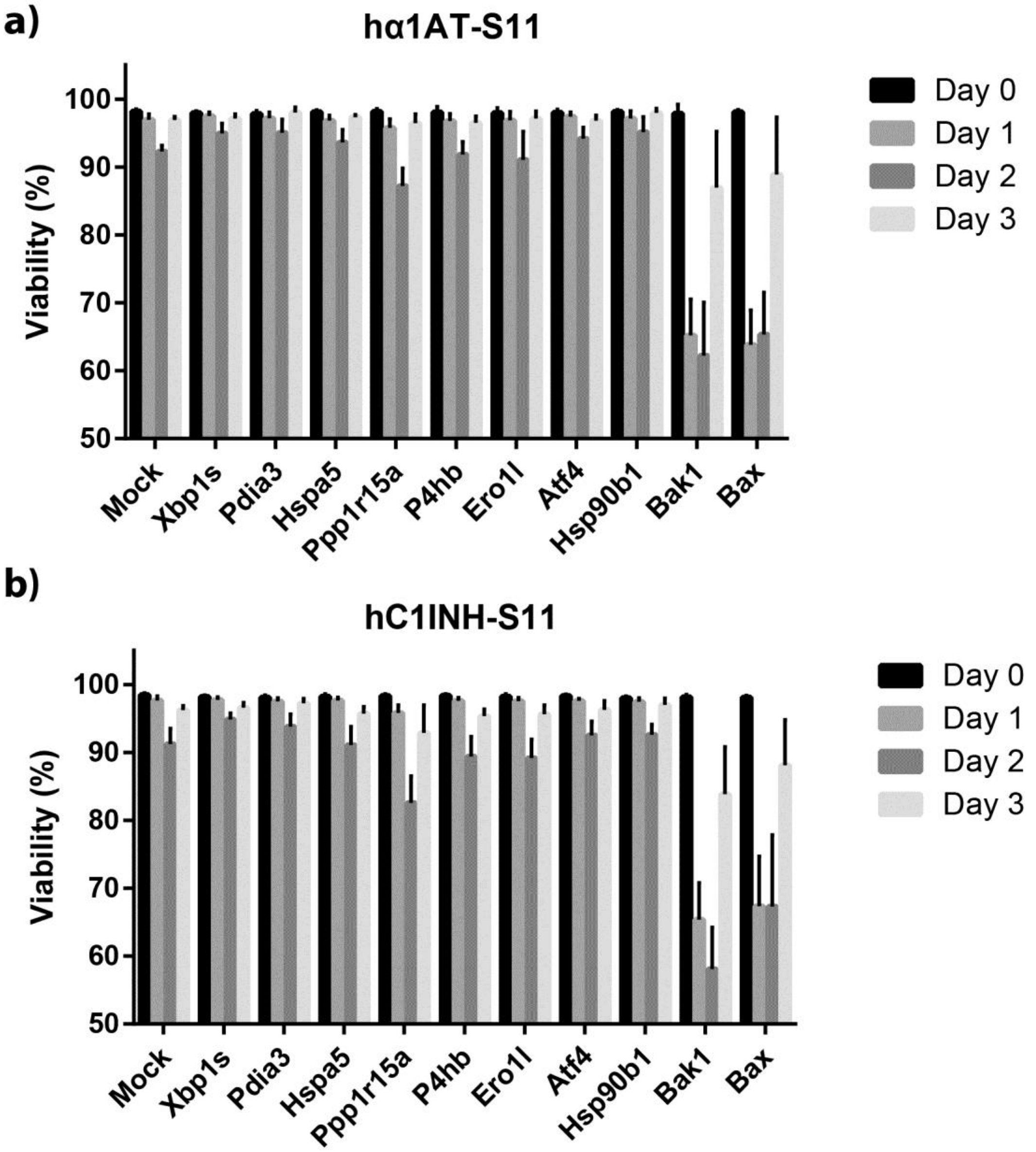

Figure S5. (a-b) Viability of cells co-transfected with S11-tagged model proteins and target genes. Mean and standard deviation are depicted (n=2; 16 wells). See Figure 5’s caption for details.

Supplementary information

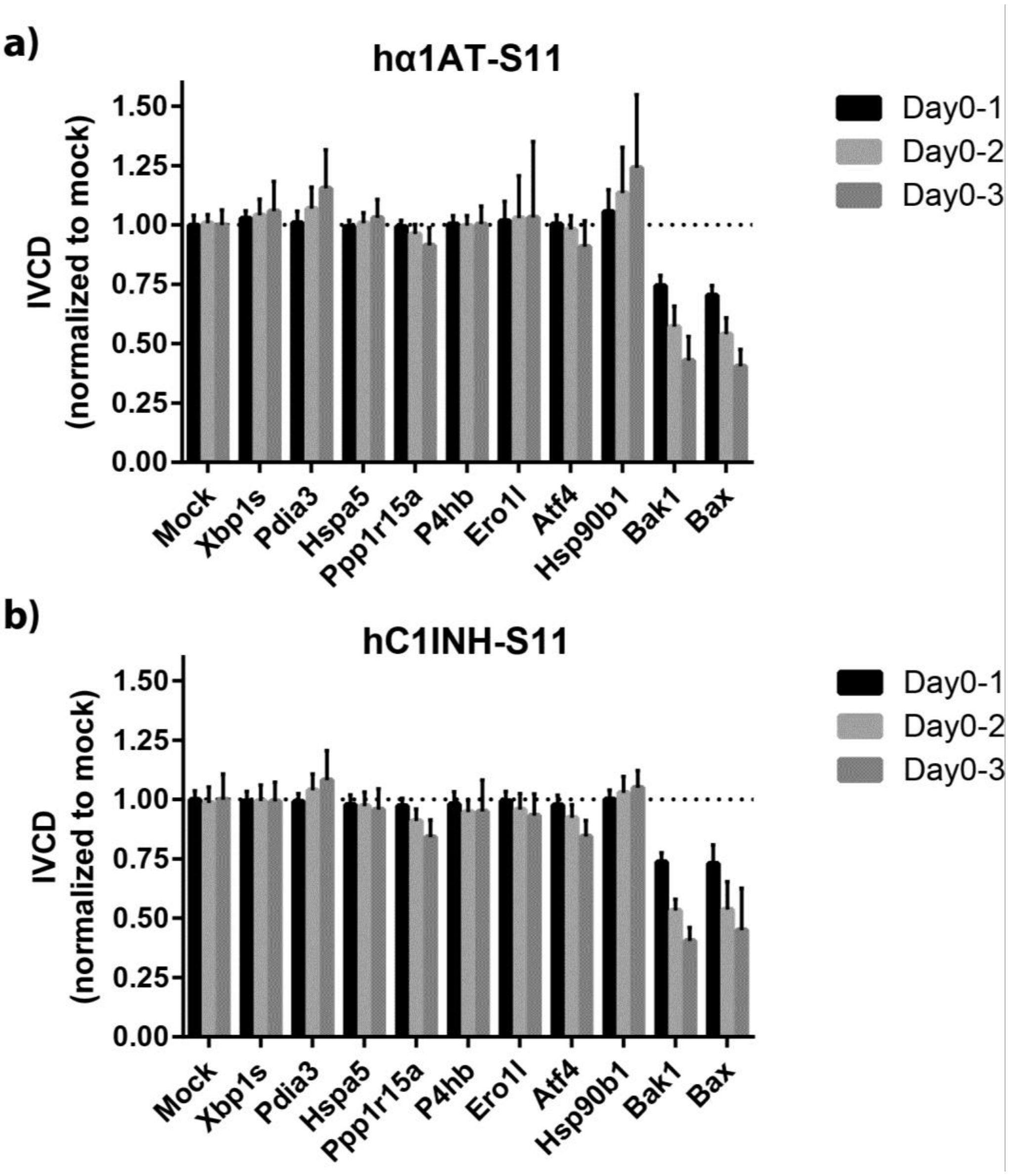

Figure S6. (a-b) Integral of viable cell density (IVCD) of cells co-transfected with plasmid encoding S11-tagged model proteins and target genes. IVCD is normalized to mock transfection (plasmid encoding model protein + mock plasmid). Mean and standard deviation are depicted (n=2; 16 wells). See Figure 5's caption for details.

Supplementary information

Supplementary Tables

Table S1: PCR primers.

Primer ID	Primer sequence (5’-3’)
23	ACTTGCGUAGTGAGTCGAATAAGGGCGACACAAA
24	AGCGACGUGAGTCGAATAAGGGCGACACCCCA
59	ACGCAAGUCAGACATGATAAGATACATTG
131	5'biotin-CTCGAGGAATTCTGCAGATATCCAGCACAG
132	GTCACGTGATCCACCTCCTGAACCTCCACCGGCTCTGGGGTCGTAGACTC
133	GAGTCGGAATTCTTATCATGTAATCCCAGCAGCATTTACGTACTCATGAAGGACCATGTGGTCACGTGATCCACCTCCTGAACCTCCACC
275	ACGTCGCUGTGAGGCTCCGGTGCCC
276	ATCGCACUTCACGACACCTGAAATGGAA
277	AGTGCGAUGCTGAGGGTTTAATTAAGTCCTCAGCAACTTGTTTATTGCAGCTTATAATGGTTAC
398	GGCCCCCAAAGTGCTACTCTTATCTGGCCAGCCCGCCTCCGGCGGCCGGGCGCTGCCGCTCATGGTACCCGGTCCGCGG
582	GGGTTTAAUATGGTGGTGGTGGCAGCGGCGCCGAGCGCGGCCACGGCGGCCCCCAAAGTG
583	ACCCTGCUGCCCGCGGACCGG
584	AGCAGGGUCGGAGGCG
585	GGACTTAAUTTAGACACTAATCAGCTGGGGGAA
586	GGGTTTAAUATGTCCATGAGGCCTGTCCC
587	AGTGGTGGUGCGGGTGGTTCTGC
590	GGGTTTAAUATGCGCTTCAGCTGCCTA
591	GGACTTAAUTTAGAGGTCCTCTTGTGCCTTC
592	GGGTTTAAUATGATGAAGTTCACTGTG
593	GGACTTAAUCTACAACTCATCTTTTTCTG
611	ACCCAGAAGUAAGCGGCCGCTCGAGTCT
612	ACTTCTGGGUGGGGTTCACGACCTTGCC
613	ACATGGTCCUTCATGAGTACGTAAATGCTGCTGGGATTACATAAGCGGCCGCTCGAGTC
616	AGCAGCAUTTACGTACTCATGAAGGACCATGTGGTCACGGGCCAGGCTCACAGGCAC
617	ACATGCAUCTGCACGAGCACGTGCACGCTCACGGCGGCACATAAGCGGCCGCTCGAGTC
619	ACGTGCACGCUCACGGCGGCACAGGTGGAGGTTCAGGAGGTGGATCAGAAGACCCCCAAGGAGACG
620	AGCGTGCACGUGCTCGTGCAGATGCATGTGGTCGTGCTTGGCCAGGCTCACAGGCAC
621	ATGCTGCUGGGATTACAGGTGGAGGTTCAGGAGGTGGATCATTGCCCGCCCAGGTGG
622	AGCAGCAUTTACGTACTCATGAAGGACCATGTGGTCACGGGCGTGCGCCGCAG
623	ATGCTGCUGGGATTACAGGTGGAGGTTCAGGAGGTGGATCAAACCCCAACGCCACCAGC
624	AGCAGCAUTTACGTACTCATGAAGGACCATGTGGTCACGGGAGGAAGCCCTGTCTCCA

Supplementary information

Primer ID	Primer sequence (5'-3')
625	ACATGCAUCTGCACGAGCACGTGCACGCTCACGGCGGCACATGAGGAATTCTGCAGATATCC
626	ATGCATGUGGTCGTGCTTTGATCCACCTCCTGAACCTCCACCGGCTCTGGGGTCGTAGAC
628	AGCGTGCACGUGCTCGTGCAGATGCATGTGGTCGTGCTTGGAGGAAGCCCTGTCTCCA
629	GGGTTTAAUATGGCATCTGGACAAGGA
630	GGACTTAAUTCATGATCTGAAGAATCTGTGTACC
631	GGGTTTAAUATGGACGGGTCCGGGG
632	GGACTTAAUTCAGCCCATCTTCTTCCAGATGG
672	GGGTTTAAUATGAGGGTCCTGTGGGTG
673	GGACTTAAUTTACAATTCATCCTTCTCTGTAGATTCC
674	GGGTTTAAUATGGCCCCGAGCCCAAG
675	GGACTTAAUTTAGCCCCGCCTCCCTCC
678	GGGTTTAAUATGCTGAGCCGTGCTTTG
679	GGACTTAAUCTACAGTTCATCCTTCACAGCTT
680	GGGTTTAAUATGGGCCGCGCCTGG
681	GGACTTAAUTCAGTGAACATTCTGTAACAAGTGCCTGAAG
700	AATACGGUTATCCACAGAATCAGGGGATAACG
701	ACCGTATUACCGCCTTTGAGTGAGCTG
721	AGGTGGAGGUTCAGGAGGTGGATCAAACCCCAACGCCACCAGC
722	ACCTCCACCUGTGCCGCCGTGAGCGTGCACGTGCTCGTG
727	ACCCTTCUGGGTGGGGTTCACGACCTTG
728	AGAAGGGUGGAGGTTCAGGAGGTGGATCACG
729	AGAAGGGUGGAGGTTCAGGAGGTGGATCAAAGC
737	GGGTTTAAUATGACCGAGATGAGCTTC
738	GGACTTAAUTTACGGAACTCTCTTCTTCC
1787	GTTGTTGCTAGCACCATGGCCAGCAGACTGACACTGCTGAC
1788	GTTGTTGAATTCTCAGGCTCTGGGGTCGTAGACTCTGC
8362	GTTGTTGAATTCCTTCTGGGTGGGGTTCACGACCTTGC
8926	GTTGTTAAGCTTACCATGCCCAGCTCCGTGAGCTGGGGCATTCTC

Supplementary information

Table S2: Coding sequences of human *Serpina1* and human *Serping1* encoding hα1AT and hC1INH, respectively. The *Serpina1* and *Serping1* genes have been codon-optimized for expression in human cells by Genewiz, Inc. Nucleotide sequences of the S11_M3-tag, S11_H7-tag and GS-linker are also shown.

	**(5’-3’)**
Serpina1<br>(signal peptide in boldface)	**ATGCCCAGCTCCGTGAGCTGGGGCATTCTCCTCCTCGCTGGCCTGTGCTGTCTGGTGCCTGTGAGCCTGGCC**GAAGACCCCCAAGGAGACGCTGCTC<br>AGAAGACAGACACATCCCACCATGACCAGGACCACCCCACCTTCAATAAGATCACCCCTAACCTCGCTGAGTTTGCCTTTTCCCTCTACAGGCAACT<br>GGCCCACCAGAGCAACTCCACCAATATCTTCTTTAGCCCTGTGAGCATCGCCACAGCCTTCGCCATGCTGAGCCTGGGCACCAAGGCTGATACACAT<br>GACGAGATCCTGGAAGGACTGAACTTCAACCTGACCGAGATCCCCGAGGCCCAGATCCACGAGGGCTTCCAGGAACTGCTGAGGACCCTGAACCAGC<br>CTGACAGCCAGCTCCAGCTCACCACCGGCAATGGCCTCTTCCTGAGCGAGGGCCTCAAGCTCGTGGATAAGTTCCTGGAAGACGTGAAGAAGCTGTA<br>CCACTCCGAAGCCTTCACAGTGAACTTTGGCGACACAGAGGAGGCCAAGAAGCAGATCAACGACTATGTGGAGAAGGGCACCCAGGGCAAGATCGTG<br>GACCTCGTGAAGGAGCTGGATAGGGACACCGTGTTCGCTCTCGTGAACTATATCTTCTTCAAGGGCAAGTGGGAGAGGCCCTTCGAGGTGAAAGACA<br>CAGAGGAAGAGGACTTCCACGTCGACCAAGTGACCACAGTCAAGGTCCCCATGATGAAGAGACTGGGCATGTTCAACATCCAGCATTGCAAAAAGCT<br>GAGCAGCTGGGTGCTGCTCATGAAGTATCTCGGCAACGCCACAGCCATCTTCTTCCTGCCCGATGAGGGCAAGCTCCAGCATCTGGAAAACGAGCTC<br>ACCCACGACATTATCACCAAGTTTCTGGAGAACGAAGACAGGAGGAGCGCTAGCCTCCACCTCCCCAAACTCAGCATCACCGGCACATATGACCTGA<br>AGTCCGTCCTCGGCCAGCTGGGCATCACAAAGGTCTTCTCCAACGGCGCCGACCTGAGCGGAGTCACAGAAGAGGCTCCCCTGAAGCTGAGCAAGGC<br>TGTGCATAAGGCCGTGCTGACAATTGACGAGAAAGGCACAGAGGCTGCCGGAGCCATGTTCCTGGAAGCTATCCCCATGAGCATCCCCCCCGAGGTG<br>AAATTCAACAAACCCTTCGTGTTCCTGATGATCGAGCAGAACACCAAGTCCCCCCTCTTCATGGGCAAGGTCGTGAACCCCACCCAGAAGTAA
Serping1<br>(signal peptide in boldface)	**ATGGCCAGCAGACTGACACTGCTGACCCTGCTGCTCCTCCTGCTGGCTGGAGACAGGGCTTCCTCC**AACCCCAACGCCACCAGCAGCAGCTCCCAGG<br>ACCCTGAGTCCCTCCAGGACAGGGGAGAAGGCAAGGTCGCCACCACCGTCATCTCCAAAATGCTCTTCGTCGAGCCCATCCTCGAGGTCAGCTCCCT<br>CCCCACCACAAACAGCACAACCAACAGCGCCACCAAGATCACCGCCAACACCACCGACGAACCCACAACCCAGCCCACCACAGAGCCTACAACACAG<br>CCTACCATCCAGCCTACCCAACCCACCACCCAGCTCCCTACCGACTCCCCTACCCAGCCTACCACAGGCTCCTTTTGTCCCGGACCTGTGACCCTGT<br>GCTCCGACCTGGAGTCCCATAGCACAGAGGCTGTCCTCGGAGATGCCCTGGTGGATTTCAGCCTCAAACTCTACCACGCCTTCAGCGCCATGAAGAA<br>GGTCGAGACCAATATGGCCTTCTCCCCCTTTAGCATCGCCAGCCTGCTCACCCAAGTCCTGCTCGGAGCCGGCGAGAATACCAAGACCAACCTGGAG<br>AGCATCCTGTCCTACCCTAAGGACTTCACCTGCGTCCACCAGGCCCTCAAGGGCTTTACCACCAAAGGAGTCACATCCGTCAGCCAGATCTTCCATT<br>CCCCTGACCTCGCCATTAGGGACACATTCGTGAACGCCTCCAGGACCCTGTACAGCAGCTCCCCTAGGGTCCTGTCCAACAACAGCGACGCCAACCT<br>GGAGCTCATTAATACATGGGTGGCCAAGAATACAAACAACAAGATTAGCAGGCTCCTGGATAGCCTGCCTTCCGACACCAGGCTCGTGCTCCTCAAT<br>GCCATCTACCTCTCCGCCAAGTGGAAGACCACATTCGACCCCAAGAAAACAAGGATGGAGCCCTTTCACTTTAAAAATAGCGTGATCAAGGTGCCCA<br>TGATGAACAGCAAGAAGTACCCTGTCGCCCACTTCATCGACCAGACCCTGAAGGCTAAGGTGGGACAGCTCCAACTGTCCCATAATCTGAGCCTGGT<br>CATCCTCGTGCCTCAGAACCTGAAGCACAGGCTGGAGGACATGGAACAGGCCCTGTCCCCCAGCGTGTTTAAGGCCATCATGGAAAAACTCGAGATG<br>TCCAAGTTTCAACCCACCCTCCTCACCCTGCCCAGAATTAAGGTCACCACAAGCCAGGACATGCTCAGCATTATGGAGAAGCTCGAGTTCTTCGATT<br>TCTCCTACGACCTCAACCTCTGCGGCCTGACAGAAGACCCTGACCTGCAGGTGAGCGCCATGCAGCACCAGACAGTGCTGGAGCTCACCGAGACAGG<br>AGTGGAAGCTGCTGCCGCCTCCGCTATTTCCGTGGCCAGGACCCTCCTGGTGTTCGAGGTGCAACAACCCTTCCTGTTCGTCCTGTGGGACCAACAA<br>CACAAGTTCCCTGTGTTCATGGGCAGAGTCTACGACCCCAGAGCCTGA
**S11_M3**	CGTGACCACATGGTCCTTCATGAGTACGTAAATGCTGCTGGGATTACA
**S11_H7**	AAGCACGACCACATGCATCTGCACGAGCACGTGCACGCTCACGGCGGCACA
**GS linker**	GGTGGAGGTTCAGGAGGTGGATCA

Supplementary information

Table S3: Commercially available plasmids and plasmids made by non-USER-based cloning.

Plasmid name	Description of plasmid	Template or source	Cloning
PL_pcDNA3.1/Zeo(+)	pcDNA3.1/Zeo(+)-backbone without insert	Plasmid obtained from Life Technologies	No cloning done
PL_hα1AT_twinstrep@Cterm	pcDNA3.1/Zeo(+)-backbone with insert encoding hα1AT with twinstrep-tag at C-terminus	pcDNA3.1/Zeo(+)::twinstrep plasmid and plasmid harboring codon-optimized CDS of *Serpina1* encoding hα1AT	PCR-amplification of *Serpina1* CDS using primers 8362 & 8926. Amplicon cut with HindIII+EcoRI and ligated into a pcDNA3.1/Zeo(+)-twinstrep vector
PL_hC1INH_notag	pcDNA3.1/Zeo(+)-backbone with insert encoding untagged hC1INH	PL_pcDNA3.1/Zeo(+) and plasmid harboring codon-optimized CDS of *Serping1* encoding hC1INH	PCR-amplification of *Serping1* CDS using primers 1787 & 1788. Amplicon cut with NheI+EcoRI and ligated into vector PL_pcDNA3.1/Zeo(+)
PL_hC1INH-S11_M3@Cterm	pcDNA3.1/Zeo(+)-backbone with insert encoding S11_M3 tagged hC1INH at the C-terminus	PL_hC1INH_notag	See text in Materials and Methods
PL_eYFP	pcDNA3/Neo-backbone with insert encoding eYFP	A kind gift from Thomas Birkballe Hansen (University of Aarhus, Denmark).	No cloning done
pGFPe_[1-10$_{OPT}$]	Bacterial expression vector encoding GFP1-10	Described in reference[1]. A kind gift from Morten Nørholm (Technical University of Denmark).	No cloning done
pBudCE4.1	pBudCE4.1-backbone without insert	Plasmid obtained from Life Technologies	No cloning done
pU0002	Backbone-vector with ampicillin resistance gene bla and pUC19 replication origin	Described in reference[4]	No cloning done
PL_Rituximab	pBudCE4.1-backbone with inserts encoding light and heavy chains of Rituximab.	Described in reference[6]	No cloning done
PL_hEPO	pcDNA3.1(+)-backbone with insert encoding codon-optimized hEPO	Described in reference[6]	No cloning done

Supplementary information

Table S4: Plasmids encoding untagged and S11-tagged model proteins constructed by USER-based cloning

Plasmid name	Features	Amplicons used
PL_hα1AT_notag	pcDNA3.1/Zeo(+)-backbone with insert encoding untagged hα1AT	1
PL_hα1AT-S11_M3@Nterm	pcDNA3.1/Zeo(+)-backbone with insert encoding S11_M3 tagged hα1AT at the N-terminus	2+3
PL_hα1AT-S11_H7@Nterm	pcDNA3.1/Zeo(+)-backbone with insert encoding S11_H7 tagged hα1AT at the N-terminus	4+5+6
PL_hα1AT-S11_M3@Cterm	pcDNA3.1/Zeo(+)-backbone with insert encoding S11_M3 tagged hα1AT at the C-terminus	7+8
PL_hα1AT-S11_H7@Cterm	pcDNA3.1/Zeo(+)-backbone with insert encoding S11_H7 tagged hα1AT at the C-terminus	7+9
PL_hC1INH-S11_M3@Nterm	pcDNA3.1/Zeo(+)-backbone with insert encoding S11_M3 tagged hC1INH at the N-terminus	10+11
PL_hC1INH-S11_H7@Cterm	pcDNA3.1/Zeo(+)-backbone with insert encoding S11_H7 tagged hC1INH at the C-terminus	12+13
PL_hC1INH-S11_H7@Nterm	pcDNA3.1/Zeo(+)-backbone with insert encoding S11_H7 tagged hC1INH at the N-terminus	15+16

Table S5: PCR amplicons used in USER-based cloning. gBlock® gene fragments were synthesized by IDT (Coralville, IA, USA), cDNA clones were obtained from OriGene Technologies Inc. (Rockville, MD, USA) and cDNA from mouse tissues was obtained from amsbio (Abingdon, UK).

Amplicon ID	Amplicon description	Fwd primer ID	Rev primer ID	Template
1	hα1AT_notag_part1of1	611	612	PL_hα1AT_twinstrep@Cterm
2	hα1AT-S11_M3@Nterm_part1of2	611	616	PL_hα1AT_twinstrep@Cterm
3	hα1AT-S11_M3@Nterm_part2of2	612	616	PL_hα1AT_twinstrep@Cterm
4	hα1AT-S11_H7@Nterm_part1of3	612	619	PL_hα1AT_twinstrep@Cterm
5	hα1AT-S11_H7@Nterm_part2of3	611	701	PL_hα1AT_twinstrep@Cterm
6	hα1AT-S11_H7@Nterm_part3of3	700	620	PL_hα1AT_twinstrep@Cterm
7	hα1AT-S11_M3/H7@Cterm_part1of2	700	727	PL_hα1AT_twinstrep@Cterm
8	hα1AT-S11_M3@Cterm_part2of2	728	701	PL_hC1INH-S11_M3@Cterm
9	hα1AT-S11_H7@Cterm_part2of2	729	701	PL_hC1INH-S11_H7@Cterm
10	hC1INH-S11_M3@Nterm_part1of2	623	701	PL_hC1INH_notag
11	hC1INH-S11_M3@Nterm_part2of2	700	624	PL_hC1INH_notag
12	hC1INH-S11_H7@Cterm_part1of2	625	701	PL_hC1INH_notag
13	hC1INH-S11_H7@Cterm_part2of2	700	626	PL_hC1INH_notag
14	hC1INH-S11_H7@Nterm_template	700	628	PL_hC1INH_notag
15	hC1INH-S11_H7@Nterm_part1of2	721	701	PL_hC1INH_notag
16	hC1INH-S11_H7@Nterm_part2of2	700	722	Amplicon #14
17	Xbp1s_part1of2	582	583	Single stranded oligo (nt 393 - 471 of refseq NM_001271730.1; primer ID 398)
18	Xbp1s_part2of2	584	585	gBlock (nt 472 – 1470 of refseq NM_001271730.1)
19	ERp57_part1of1	590	591	cDNA clone (#MC200134)

20	BiP_part1of1	592	593	cDNA clone (#MC202686)
21	Bak1_part1of1	629	630	gBlock (nt 254 – 883 of refseq NM_007523.2)
22	Bax_part1of1	631	632	gBlock (nt 124 – 702 of refseq NM_007527.3)
23	Hsp90b1_part1of1	672	673	Mouse cDNA (#C4334566)
24	GADD34_part1of1	674	675	Mouse cDNA (#C4334566)
25	PDI_part1of1	678	679	Mouse cDNA (#C4334566)
26	Ero1α_part1of1	680	681	Mouse cDNA (#C4334566)
27	ATF4_part1of1	737	738	Mouse cDNA (#C4334566)
28	FAST1_EF-1α_FAST2	275	276	pBudCE4.1
29	FAST2_PacI/Nt.BbvCI USER cassette_SV40pA_FAST6	277	59	PL_pcDNA3.1/Zeo(+)
30	FAST6_ backbone_FAST1	23	24	pU0002

Table S6: PL_TGExpr and plasmids encoding target genes (from mouse) constructed by inserting CDS into the ‘PacI/Nt.BbvCI A’ USER-cassette of PL_TGExpr (see Supplementary Fig. S1b).

Plasmid name	Features	Gene name	Protein name	Uniprot ID	Amplicons used
PL_TGExpr	pU0002-backbone harboring EF1α_PacI/Nt.BbvCI A-sv40pA	-----------------	--	-----------------	28+29+30
PL_MmXbp1s	PL_TGExpr:: murine Xbp1s	*Xbp1s*	XBP1-S	O35426-2	17+18
PL_MmPdia3	PL_TGExpr:: murine Pdia3	*Pdia3*	Protein disulfide-isomerase A3 (ERp57)	P27773-1	19
PL_MmHspa5	PL_TGExpr:: murine Hspa5	*Hspa5*	GRP-78 (BiP)	P20029-1	20
PL_MmBak1	PL_TGExpr:: murine Bak1	*Bak1*	Bak1 protein	O08734-1	21
PL_MmBax	PL_TGExpr:: murine Bax	*Bax*	Apoptosis regulator BAX	Q07813-1	22
PL_Mm Ppp1r15a	PL_TGExpr:: murine Ppp1r15a	*Ppp1r15a*	Protein phosphatase 1 regulatory subunit 15A (GADD34)	P17564-1	23
PL_Mm P4hb	PL_TGExpr:: murine P4hb	*P4hb*	Protein disulfide-isomerase (PDI)	P09103-1	24
PL_MmEro1l	PL_TGExpr:: murine Ero1l	*Ero1l*	ERO1-like protein alpha (Ero1α)	Q8R2E9-1	25
PL_MmAtf4	PL_TGExpr:: murine Atf4	*Atf4*	Cyclic AMP-dependent transcription factor ATF-4	Q06507-1	26
PL_MmHsp90b1	PL_TGExpr:: murine Hsp90b1	*Hsp90b1*	Endoplasmin (GRP-94)	P08113-1	27

Supplementary information

Table S7: Doubling time (h) from day 0-3 in a 96-HDW microplate (96 wells) calculated by exponential growth equation (least squares fit) in GraphPad Prism. See legend for Figure 3 for details.

	1	2	3	4	5	6	7	8	9	10	11	12
A	19.0	18.7	19.8	19.9	19.9	20.0	20.3	18.9	19.3	19.7	19.3	18.6
B	18.3	20.5	20.9	19.8	20.5	19.5	18.3	18.9	19.1	19.9	19.2	18.6
C	20.0	20.2	20.1	19.3	19.5	19.6	19.4	19.3	20.1	18.9	19.1	20.4
D	19.9	19.2	20.2	20.1	19.3	18.8	18.6	20.4	19.6	19.5	19.5	17.9
E	19.0	18.6	17.9	18.3	18.0	18.8	19.6	18.7	18.6	18.9	18.8	17.8
F	18.9	20.6	19.3	18.9	18.3	20.4	19.3	19.2	18.9	20.0	19.0	18.3
G	19.6	18.4	19.5	18.9	20.1	18.9	20.0	19.0	19.2	18.9	18.6	18.1
H	19.0	18.9	19.6	20.0	19.5	19.1	20.3	18.3	18.8	19.6	17.9	18.1

Table S8: Coefficient of correlation (R^2) of fit to exponential growth equation. See legend for Figure 3 for details.

	1	2	3	4	5	6	7	8	9	10	11	12
A	0.9995	0.9987	0.9997	0.9988	0.9979	0.9997	0.9995	0.9998	0.9998	0.9999	1	0.9991
B	0.9975	1	0.9989	0.9996	1	0.9997	0.9991	0.9998	0.9999	1	0.9997	0.9995
C	0.9999	0.9999	0.998	0.9988	0.9998	0.9997	0.9969	0.9992	0.9989	0.9996	0.9994	0.9996
D	0.9999	0.9995	0.9997	0.9996	0.9997	0.9996	0.9985	1	0.9999	0.9997	0.9983	0.9989
E	0.9995	0.9996	0.9968	0.9982	0.9975	0.9998	0.9997	0.9984	0.9987	0.9996	1	0.9988
F	0.9991	0.9997	0.9995	0.9991	0.9986	0.9999	1	0.9999	1	0.9998	1	0.9998
G	0.9995	0.9978	0.9982	0.9997	0.9995	0.9997	0.9999	0.9991	1	1	0.9999	0.9995
H	0.9997	1	0.9993	0.9997	0.9998	0.9999	1	0.9991	0.9993	0.9997	0.9995	0.9988

Table S9. Comparison of relative titer and relative q_p of untagged and S11-tagged hα1AT and hC1INH. See details in legends for Figure 5 and 6. Titers from ELISA quantification (Fig. 5e-f and Fig. 6a-b) are used.

	**hα1AT: Mock vs. *Atf4***			**hC1INH: Mock vs. *Xbp1s***		
	**hα1AT untagged (fold-change)**	**hα1AT-S11 (fold-change)**	**Difference (%)**	**hC1INH untagged (fold-change)**	**hC1INH-S11 (fold-change)**	**Difference (%)**
**Titer**	1.23	1.36	+11	1.26	1.46	+16
q_p	1.32	1.51	+15	1.36	1.47	+8

Supplementary information

Supplementary References

1. Toddo, S. *et al.* Application of split-green fluorescent protein for topology mapping membrane proteins in Escherichia coli. *Protein Sci.* **21,** 1571–6 (2012).

2. Cabantous, S. & Waldo, G. S. In vivo and in vitro protein solubility assays using split GFP. *Nat. Methods* **3,** 845–54 (2006).

3. Cabantous, S., Terwilliger, T. C. & Waldo, G. S. Protein tagging and detection with engineered self-assembling fragments of green fluorescent protein. *Nat. Biotechnol.* **23,** 102–7 (2005).

4. Hansen, B. G. *et al.* Versatile enzyme expression and characterization system for Aspergillus nidulans, with the Penicillium brevicompactum polyketide synthase gene from the mycophenolic acid gene cluster as a test case. *Appl. Environ. Microbiol.* **77,** 3044–3051 (2011).

5. Lund, A. M. *et al.* A versatile system for USER cloning-based assembly of expression vectors for mammalian cell engineering. *PLoS One* **9,** e96693 (2014).

6. Kol, S., Kallehauge, T. B., Adema, S. & Hermans, P. Development of a VHH-Based Erythropoietin Quantification Assay. *Mol. Biotechnol.* **57,** 692–700 (2015).
